# In Situ Measured Tooth Flank Wear of Plastic Gears under Spectrum Loading

**DOI:** 10.3390/polym14235239

**Published:** 2022-12-01

**Authors:** Christoph Herzog, Michael Wolf, Dietmar Drummer

**Affiliations:** Institute of Polymer Technology (LKT), Friedrich-Alexander-University Erlangen-Nuremberg, 91058 Erlangen, Germany

**Keywords:** in situ, gear testing, plastic gears, gear wear, gear design

## Abstract

The wear behaviour of PBT-steel gear sets under temporarily changed load has been investigated using an in situ gear test rig developed at the LKT. The in situ test method is based on analysing the timing differences between the index pulses of rotary encoders on the input and output shaft of the test rig. The loading torque was varied between two levels and compared to the permanently applied equivalent average load in terms of the resulting tooth flank wear. Moreover, the number of load changes has been varied to analyse the influence of load changes on the gear wear. The results show that the applied load spectrum determines the resulting tooth flank wear even if the average applied load is the same. Moreover, it could be shown that the sequence of the applied load, i.e., the load history, plays an important role, since the applied load and the duration of the applied load within the run-in-stage disproportionately affect the wear behaviour over time.

## 1. Introduction

Gears are widely used in power transmission systems in various industries and machines [1]. Compared to metal gears, plastic gears show many advantageous characteristics, such as dry run capability [2], being lightweight [3], noise reduction [1], and low manufacturing costs [2] due to economic mass production by injection moulding. On the contrary, the properties of plastic gears lead to specific failure modes. Plastic gears mainly fail due to root fracture, melting, and tooth flank wear [4]. Since tooth flank wear influences the functionality and quality of the product [5], detailed knowledge about the wear behaviour of plastic gears is required to design gearboxes for specific applications [6]. In most applications, gears operate under variable load [7]. Consequently, the design of plastic gears in terms of wear should consider the applied load spectrum. However, current design guidelines such as VDI 2736 [8] and most studies in the literature only consider single stage loading. Therefore, this work presents a detailed investigation of the tooth flank wear of plastic gears under variable loading using an in situ gear test rig.

### 1.1. Wear Behaviour of Plastic Gears

**Figure 1:**
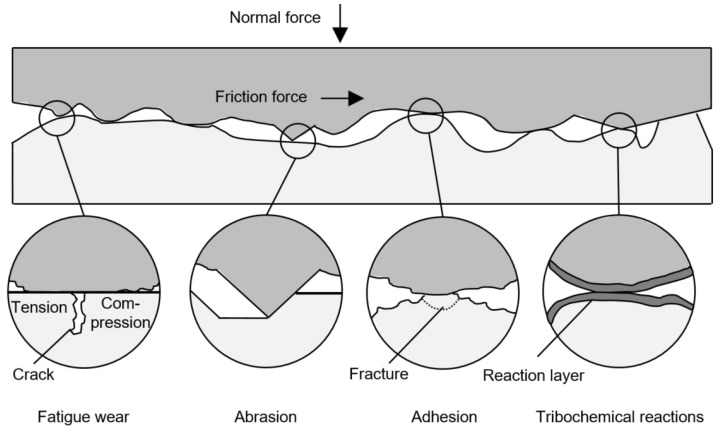
Wear mechanisms of tribological systems according to [10].

Four different wear mechanisms, based on physical and chemical interactions in the contact area of tribological systems, exist (Figure 1): fatigue wear, abrasion, adhesion, and tribochemical reactions. These mechanisms cause material and geometrical changes of the micro-contacts depending on the structure of the tribological system and the load collective. The relevant load parameters of wear processes are the normal force, the velocity, the temperature, and the loading duration [9].

Fatigue wear is caused by the formation of cracks in surface areas as a result of varying tribological loads. Scratching and cutting loads lead to abrasion wear. Adhesion occurs due to a separation of contact surfaces [9], which have been joined by galling in the case of steel/steel contacts [10]. According to Faatz [11], polymers show lower secondary valence forces than steel, which leads to less adhesion in polymer/steel contacts. Especially pairings of the same polymers show the most adhesion [11]. If the tribological load favours chemical reactions between the materials in contact or the lubricant, which lead to the formation of reaction layers or particles of differing wear rates, the underlying wear mechanism is a tribochemical reaction [9].

In most tribological systems, there is a superposition of different wear mechanisms [10]. However, gears are characterised by a Hertzian contact pressure and a periodically applied load. Therefore, the fatigue wear can be particularly pronounced [12].

Fatigue wear of polymer gears is caused by stress concentrations beneath micro-contacts of the polymer surface due to friction forces [13]. These stress concentrations result in the formation and propagation of cracks, which subsequently coalesce [14]. Eventually, flat wear debris parts break away from the surface [11].

The wear behaviour of plastic gears is influenced by various factors. According to Feulner [2], the rotational speed, as well as the loading torque, influence the gear wear. However, the effect of increasing rotational speed on the gear wear is much lower and, thus, less relevant compared to the effect of the applied loading torque. Moreover, the wear behaviour is temperature-dependent [11]. Investigations of acetal gears [15] show a sudden rise in gear wear at a critical transmitting torque because, when the surface temperature reaches the material‘s melting point, this is observed.

### 1.2. Initial Wear Behaviour of Polymer/Steel Contacts

Polymer/steel gear pairings typically show a degressive wear behaviour over time. After an initial phase characterised by a high wear rate, a steady phase with a lower linear wear rate follows [2]. This is due to the transition of the less wear-resistant outer zone to the more crystalline and, thus, more wear resistant inner zone of injection moulded parts [16]. Moreover, within the initial phase, an alignment of the surfaces in the contact area and the formation of wear-reducing interlayers takes place, which reduces the further wear rate [17].

The run-in wear behaviour of polymer/steel gear pairings is dependent on the surface structure and the surface roughness of the steel gear. However, there is no direct correlation between the tooth flank temperature and the initial wear behaviour of the plastic gear. [18]

### 1.3. Operating Properties of Gears under Spectrum Loading

There are numerous studies in the field of lifetime prediction of parts subjected to varying loads. Different fatigue damage models are proposed in the literature. Since none of these models considers all the relevant factors, such as load dependence, multiple damage stages, nonlinear damage evolution, load sequence and interaction effects, overload effects, small amplitude cycles below fatigue limit, and mean stress, there is no model of general acceptance [19]. Therefore, the Miner linear damage rule [20], based on the concept of Palmgren [21], is commonly used for the design of parts subjected to varying loads. The Miner rule is based on the assumption that the energy accumulation per cycle leads to a linear damage summation and eventually to failure of the part. The applied load spectrum is expressed as load stages of different amplitudes with a frequency *n_i_*. The damage for every load stage *r_i_* is calculated using the maximum number of load cycles of the S–N fatigue curve *N_i_* (Equation (1)):

(1) $$r_{i}=\frac{n_{i}}{N_{i}}$$

The damage of the load stages is accumulated resulting in the total damage *D* using Equation (2):

(2) $$D=\sum^{\;}r_{i}=\sum^{\;}\frac{n_{i}}{N_{i}}$$

Failure of the part occurs according to Miner when the theoretical lifetime is exceeded at *D* = 1.

Other models refer to different physical reasons for failure, such as endurance limit changes (Henry [22]), number of damaged nuclei (Corten/Dolan [23]), and crack initiation and propagation (Grover [24]).

Although thermoplastic parts fail similarly to metal parts by fracture under cyclic load, the underlying process at the microscale is different. Since the surface and inner structure of polymers are not perfectly homogenous, varying load is not exclusively stored elastically. Instead, the cyclic applied load leads to non-reversible stretching in the inhomogeneous zones. The stretching causes heat generation, which favors the mobility of the polymer molecules. Eventually, the polymer fails due to breakage [25]. Moreover, thermoplastic parts can fail due to rate-dependent thermal failure since the damping properties of polymers lead to a dissipation of the applied energy as heat [26].

As far as gear sets under varying load are concerned, numerous studies exist in the literature. Their focus mostly lies on lifetime prediction, condition monitoring, and experimental lifetime testing of metal gear sets. Since the lifetime of gears is shorter under varying loads [27], there are different studies focusing on the optimization of lifetime prediction under varying loads. Lifetime predictions are performed by running simulations [27], numerical models of bevel gears in terms of multiaxial fatigue [28], and thin-rim spur gears in terms of bending fatigue [29]. The results of the studies mentioned above are improved lifetime prediction models. Hein et al. [30] follow a more experimental approach for the lifetime prediction of steel gears in terms of relevant damage mechanisms such as tooth root breakage and pitting by designing a load spectrum truncation for accelerated lifetime testing. Yang [31] investigated different linear cumulative damage hypotheses for gears. The gear tests under spectrum loading show the best results for the Corten/Dolan hypothesis. However, the results also show a discrepancy to the experimental lifetimes in higher reliability range. Therefore, progressive damage accumulation rules should be used.

There are considerably less studies on polymer gears under spectrum loading. For accelerated gear testing, step loading tests were performed by Pogačnik and Tavčar [32] and Mao et al. [33]. In this case, the test gears are loaded with a constant torque for a certain time period. After this period, the load is increased for another time period. Mao [33] repeated this procedure until failure of the polymer gear. The results show a distinct rise of the wear rate when a critical temperature is caused at certain torques.

In summary, there are various studies of metal gears under varying torques and few studies of polymer gears. However, the focus is on lifetime testing and prediction and not on experimental investigations of the tooth flank wear of plastic gears under application-oriented load conditions such as load changes. Therefore, this work provides a detailed investigation of polymer/steel gear pairings under spectrum loading using an in situ gear test rig of the LKT, which enables the measurement of the tooth flank wear continuously during the test run. The aim of this study is to analyse the interactions between varying loading torques, the resulting temperatures, and the formation of wear debris of polymer/steel gear pairings in detail.

## 2. Materials and Methods

The wire cut pinions used for this work were made of 100Cr6. For the plastic gears PBT, type Pocan B1305 by the LANXESS AG (Cologne, Germany), which is a typical material for gears in actuating drives, was used. The technical specifications of the investigated gear pairing according to DIN 867 [34] is shown in Table 1.

The plastic gears were injection-moulded according to the processing data sheet using an Arburg 370U-700-30-30 injection-moulding machine by Arburg GmbH Co. KG (Loßburg, Germany). The most important processing parameters are shown in Table 2. The material has been dried at 120 °C for 6 h before processing.

**Figure 2:**
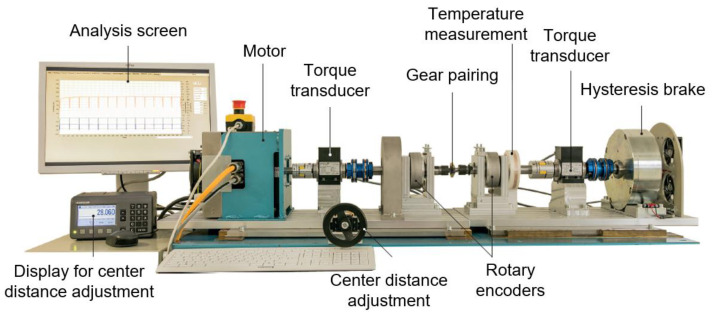
Main components of the in situ gear test rig of the LKT [6].

The gear tests were performed on a in situ test rig (Figure 2), which enables the measurement of the tooth deformation and flank wear during the run. A three-phase A.C. motor, type DSM150N, by Baumüller, Nuremberg, Germany, on the input side, drives the steel pinion. The loading torque is applied on the plastic gear by a hysteresis brake, type CHB-12, by Magtrol, Rossens, Switzerland on the output side. Torque fluctuations during the test run and the according frequency spectrum are measured by torque transducers, type TMB307, by Magtrol, Rossens, Switzerland, on the input and the output side. The temperature of the plastic gear is measured during the run by positioning a thermocouple, type K, in a drilled hole with a diameter of 0.6 mm in the tooth root. The temperature information is transferred to a data logging PC via telemetry, type TEL1-PLM-IND by Kraus Messtechnik GmbH, Otterfing, Germany. The continuous tooth wear and deformation measurement is based on rotary encoders, type A020 by Fritz Kübler GmbH, Villingen-Schwenningen, Germany, on the input and output shaft. The functional principle of the in situ gear test rig has already been validated in terms of the measurement of the tooth flank wear and plastic deformation [35].

**Figure 3:**
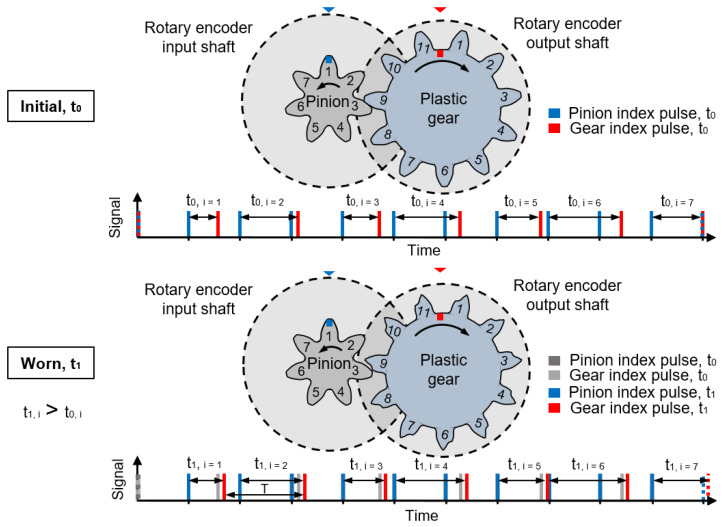
Tooth wear induced change of the timing difference between input and output index pulses.

In order to measure the tooth flank wear and deformation of the plastic gear on the output side, the index pulses of the rotary encoders on the input and output shaft are used. The timing difference between the index pulses increases during the test run (t_0_ < t_1_) because the index pulse of the plastic gear delays with increasing wear and deformation (Figure 3).

**Figure 4:**
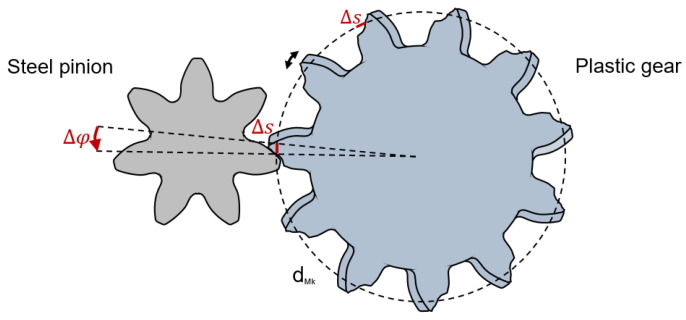
Tooth wear induced angular displacement between pinion and gear.

The change of the timing difference Δt = t_1_ − t_0_ is an expression of the angular displacement between the input and output shaft. The displacement between gear and pinion due to wear is shown in Figure 4.

Since T is the time for one rotation of the plastic gear on the output shaft, Equation (1) can be used to calculate the angular displacement Δφ between pinion and gear:

(3) $$∆\varphi \;=\frac{∆t}{T}\;\cdot \;360{}^{\circ}$$

The tooth flank wear Δs can be determined using the angular displacement Δφ according to Equation (2), at the measuring diameter d_Mk_ = 38.146 mm according to DIN 3977 [36]:

(4) $$∆s\;=\;\Delta \varphi \frac{\pi \cdot \;d}{360{}^{\circ}}\;$$

At the given sampling rate of 80 MHz, the encoders can sample every 0.0000000125 s which equals a 0.00003° rotation. At a rotational speed of 1000 min^−1^, this results in a tooth deformation measurement of ±0.01 µm, assuming a polymer gear with a pitch diameter of 39 mm is tested. Thus, the resolution of the test rig is very high, but decreasing with higher rotational speeds.

**Figure 5:**
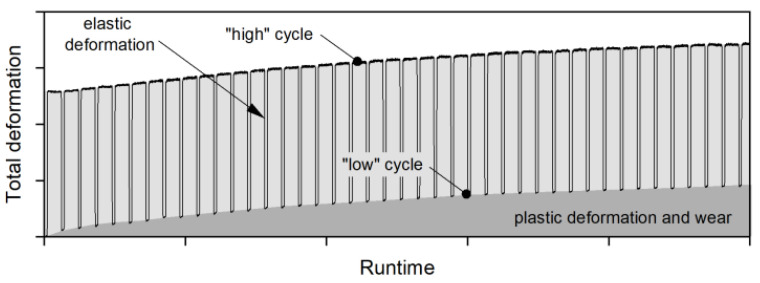
Distinction between elastic deformation and plastic deformation and wear, schematically [37].

The test rig enables switching between a high testing torque and a low measuring torque after a predefined numbers of load cycles. One load cycle is defined as the time period until every tooth of the pinion and the gear has been in contact. By switching between the high and low torque the elastic component of the total deformation is removed. Thus, the remaining deformation (plastic deformation and wear), can be evaluated as seen in Figure 5.

**Figure 6:**
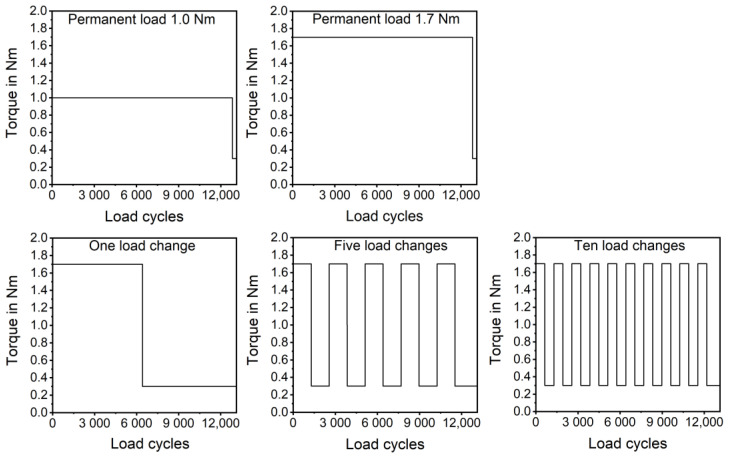
Torque settings used for the gear tests.

In this work, the influence of load changes on the tooth flank wear has been investigated. For this reason, the loading torque was changed between 1.7 Nm and 0.3 Nm (measuring torque). The measuring torque has to be as low as possible to remove most of the elastic deformation. At the same time, the measuring torque has to be high enough to ensure that the gears do not lose contact. Therefore, 0.3 Nm has been chosen. The load has been changed one, five, and ten times during the test run, and the resulting wear has been compared to the equivalent permanent load of 1.0 Nm and a maximum permanent load of 1.7 Nm. At the end of the test runs, the torque was reduced to 0.3 Nm for 300 load cycles in order to ensure comparability. The used torque settings are shown in Figure 6. For each torque setting, a repetition number n of 3 gears has been tested.

The gear tests were performed at a rotational speed of 1170 min^−1^. The temperature of the plastic gear has been recorded continuously. The test duration was 5 × 10^5^ pinion rotations, which equals 12.820 load cycles, or 7.17 h at the given speed. Since the run-in stage is already completed after about 3 h, the wear only increases linearly at a lower wear rate. Therefore, the chosen test duration of 7.17 h seems to be sufficient to analyse the wear behaviour.

A dynamic mechanical analysis of the used material, PBT Pocan B1305, was performed to correlate the resulting temperatures of the gears during the test run with the mechanical properties of the material. For this purpose, an ARES-rheometer, TA Instruments, New Castle, US was used. The chosen load type for the analysis was torsion, and the heating rate was 2 K/min.

To investigate the influence of the load history on the gear wear, additional gear tests with a repetition number n of 3 gears were performed. These tests were conducted with one load change but starting with the low torque of 0.3 Nm for 6410 load cycles and a following torque of 1.7 Nm for the remaining 6410 load cycles. To quantify the gear wear in detail, the contour of the gears has been measured using a coordinate measurement machine, type Leitz PMM 654, by Hexagon Metrology GmbH, Wetzlar, Germany, with a tip radius of 1.0 mm. The contours have been compared to the average untested contour measured via coordinate measurement machine. The tooth flank wear was evaluated as the biggest normal distance between the average untested contour and the average contour after the test run.

## 3. Results and Discussion

**Figure 7:**
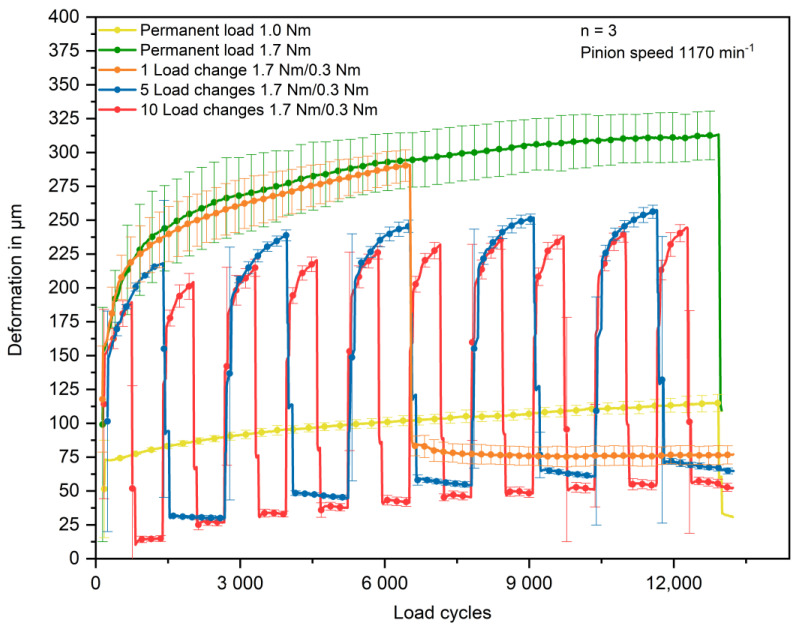
In situ measured gear wear in dependence of the applied load spectrum.

The gear tests show that load changes lead to higher wear compared to the equivalent permanent load of 1.0 Nm, which is 30.7 µm ± 4.2 µm after the defined test duration. The in situ measured wear increases with less load changes. At ten load changes, the wear is 52.4 µm ± 3.2 µm. It further increases from 64.8 µm ± 2.4 µm at five load changes to 77.1 ± 6.5 µm at one load change. The highest wear of 109.4 µm ± 8.2 µm is measured at the gears tested at 1.7 Nm permanent load. It has to be noted that the total deformation of the gears tested with 1.7 Nm permanent load and one load change is higher at the beginning of the rest run than the deformation of the gears tested with five and ten load changes. However, the differences are still within the range of the standard deviations. The in situ measured results of the gear tests are summarised in Figure 7

**Figure 8:**
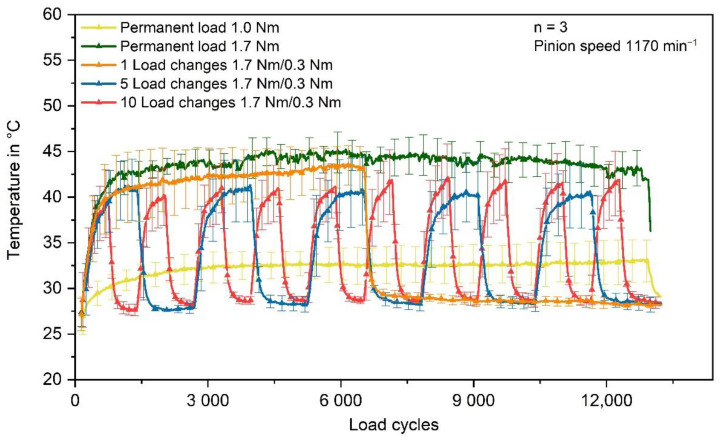
In situ measured tooth root temperature of the polymer gear.

One explanation for the lower wear at 1.0 Nm permanent load is the resulting temperature being lower than at varying torques. The average equilibrium state temperature at 1.0 Nm permanent torque is about 33 °C ± 2 °C. When the loading torque is changed, the temperature rises higher. For five and ten load changes, the maximum temperature is about 41 °C ± 3 °C. In the case of one load change, the temperature even rises up to 43 °C ± 2 °C, which is only about 2 °C lower than the equilibrium temperature at 1.7 Nm permanent torque (45 °C ± 2 °C). The temperatures during the gear test runs, measured via thermocouple in the tooth root area, are shown in Figure 8.

The maximum reached temperatures in the case of one, five, and ten load changes do not vary distinctly. Moreover, the temperature of the reference test runs at 1.7 Nm permanent load is only slightly higher. In general, the different torque settings are causing temperatures which differ mostly within the range of the standard deviations. Therefore, temperature might play a negligible role for the different wear behaviour under varying load spectrums of the same average load. However, the dynamic mechanical analysis shows that the glass transition starts at about 40 °C and ends at about 60 °C, with decreasing storage modulus G’. Since the stored reversible energy decreases, the wear rate increases.

The resulting wear shown in Figure 7 implies that the applied load and the duration of the applied load in the run-in phase is crucial for the resulting wear. Considering the wear mechanisms explained in Section 1, the reason could be that high torques in the run-in phase cause more stress concentrations, since the alignment of the surfaces has not been completed. This may result in more pronounced crack initiation and propagation.

In the case of one load change, the polymer gear is loaded with the high torque of 1.7 Nm during the vast majority of the run-in stage. However, with more load changes, the loading period with the high torque of 1.7 Nm converges to only 50% of the run-in phase and, therefore, results in lower wear.

**Figure 9:**
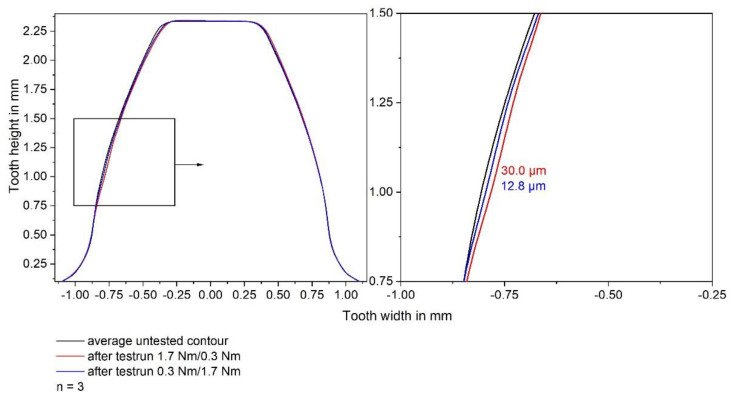
Coordinate measurements of gears tested with one load change in different sequence.

In order to verify this hypothesis, gear tests have been performed with one load change but starting with the low torque of 0.3 Nm for 6410 load cycles and following with a rise to 1.7 Nm loading torque for the remaining 6410 load cycles. In this case, the loading torque is low for the majority of the run-in phase. The low loading torque within the run-in phase should enable an alignment of the surfaces, leading to less stress concentrations and less crack initiation and propagation, and, thus, less wear, when the high loading torque is applied. The results of the coordinate measurements are shown in Figure 9.

The coordinate measurements show almost exclusively tooth flank wear. At the tooth tip of the side not in contact with the pinion minor plastic deformation can be seen. The tooth flank wear of the side in contact is not distributed uniformly. Wear mainly occurs in the tip area and below the pitch point with the greatest amount of wear being observed below the pitch point. The average contour of the gears tested with the low torque at the start of the test run shows much less wear, which supports the hypothesis mentioned above. However, the wear measured via the coordinate measurement machine is 12.8 µm for the test runs, with the low torque at the start, and 30.0 µm for the test runs with the high torque at the start. These values are much lower than the in situ measured tooth flank wears of 45.4 ± 3.5 µm (0.3 Nm/1.7 Nm loading torque) and 77.1 ± 6.5 µm (1.7 Nm/0.3 Nm loading torque). The deviation between in situ and ex situ measurements has already been analysed in a previous article by Herzog et al. [35]. The results show good correlation of in situ and ex situ measurements. However, the measured values are not the same since the in situ test rig measures timing differences during operation, and the coordinate measurement compares contours locally. Nevertheless, the Pearson correlation coefficient of 0.9818 indicates that the in situ gear test rig is qualified for measuring the gear wear and plastic deformation continuously.

**Figure 10:**
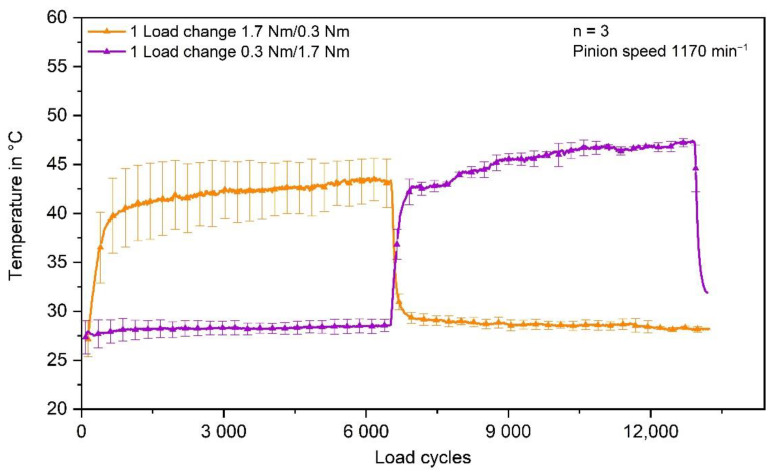
Temperature of the gears tested with one load change in different sequence.

The measured temperature of the gears tested with one load change in different sequence is shown in Figure 10. The maximum tooth root temperature of the gears loaded with 0.3 Nm first is about 47 °C ± 1 °C, whereas the maximum temperature of the gears loaded with 1.7 Nm first is about 43 °C ± 2 °C.

This confirms the assumption that temperature is not the main reason for the different wear behaviour under varying loads. Probably the differences in temperature and correspondingly the storage modulus between the different torque settings are not significant enough to influence the wear behaviour distinctly.

However, additional gear tests with longer test durations until failure of the plastic gear have to be performed to confirm the results and come to definitive conclusions.

## 4. Conclusions

In practical applications, gears are subjected to load collectives rather than single stage loading. Since most of the current wear investigations of polymer gears only consider single stage loading, this work provides a more comprehensive approach by testing PBT-steel gear pairings under spectrum loading regarding the resulting gear wear.

The results show that load changes lead to higher wear than the permanently applied average equivalent load. Moreover, the wear is increasing with a decreasing number of load changes. One explanation is that the load changes lead to higher peak temperatures causing more wear since the storage modulus decreases with increasing temperatures within the observed range. However, coordinate measurements give evidence that the applied load and the duration of the applied load during the run-in stage determine the resulting wear, since higher loads forward crack initialisation and propagation when the contact surfaces are not aligned due to more pronounced stress concentrations.

Future research should be conducted in the field of application-oriented gear wear testing in order to improve current design guidelines such as VDI 2736. Therefore, further research is currently conducted at the LKT to examine the wear behaviour of polymer/steel gear sets under temporarily varying torques and speeds. Especially longer test durations until failure of the gear and additional load spectra have to be investigated in the future.

## Figures and Tables

**Table 1:** Technical specifications of the investigated gear set.

	DIN 867	Pinion	Gear
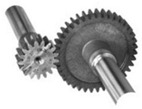	Material	100Cr6	PBT Pocan B1305
	Module	1 mm	
	Pressure angle	20 °	
	Number of teeth	17	39
	Gear width	8 mm	6 mm
	Profile shift	0.2045 mm	−0.3135 mm

**Table 2:** Main processing parameters for the manufacturing of the plastic gears.

Processing Parameter	Parameter Setting
Screw diameter	18 mm
Mass temperature	260 °C
Mould temperature	90 °C
Injection/Holding/Cooling/Cycle time	2.2 s/6 s/25 s/42.8 s
Holdingpressure	600 bar
Cylinder temperature profile (Nozzle → indentation	260 °C/250 °C/240 °C/230 °C/90 °C

## Data Availability

Not applicable.
